# High Plasma Levels of Islet Amyloid Polypeptide in Young with New-Onset of Type 1 Diabetes Mellitus

**DOI:** 10.1371/journal.pone.0093053

**Published:** 2014-03-26

**Authors:** Johan F. Paulsson, Johnny Ludvigsson, Annelie Carlsson, Rosaura Casas, Gun Forsander, Sten A. Ivarsson, Ingrid Kockum, Åke Lernmark, Claude Marcus, Bengt Lindblad, Gunilla T. Westermark

**Affiliations:** 1 Department of Clinical and Experimental Medicine, Division of Pediatrics and Diabetes Research Centre, Linköping University Hospital, Linköping, Sweden; 2 Department of Pediatrics, Lund University Hospital, Lund, Sweden; 3 Department of Pediatrics, the Queen Silvia Children's Hospital, Gothenburg, Sweden; 4 Department of Pediatrics, University Hospital MAS, Malmö, Sweden; 5 Department of Molecular Medicine, Karolinska Institute, Stockholm, Sweden; 6 Department of Clinical Sciences, Lund University, Malmö, Sweden; 7 Department of Pediatrics, Karolinska University Hospital, Huddinge, Sweden; 8 Department of Medical Cell Biology, Uppsala University, Uppsala, Sweden; University of Michigan Medical School, United States of America

## Abstract

**Aims/Hypothesis:**

Islet amyloid polypeptide (IAPP) is a beta cell hormone secreted together with insulin upon glucose stimulation. IAPP participates in normal glucose regulation, but IAPP is also known for its ability to misfold and form islet amyloid. Amyloid fibrils form through smaller cell toxic intermediates and deposited amyloid disrupts normal islet architecture. Even though IAPP and amyloid formation are much discussed in type 2 diabetes, our aim was to study the significance of IAPP in type 1 diabetes.

**Results:**

Plasma IAPP levels in children and adolescents with newly diagnosed type 1 diabetes (n = 224) were analysed and concentrations exceeding 100 pmol/L (127.2 – 888.7 pmol/L) were found in 11% (25/224). The IAPP increase did not correlate with C-peptide levels.

**Conclusions/Interpretation:**

Plasma levels of IAPP and insulin deviate in a subpopulation of young with newly-diagnosed type 1 diabetes. The determined elevated levels of IAPP might increase the risk for IAPP misfolding and formation of cell toxic amyloid in beta cells. This finding add IAPP-aggregation to the list over putative pathological factors causing type 1 diabetes.

## Introduction

Type 1 diabetes (T1D) results from a chronic autoimmune destruction of the pancreatic beta cells and accounts for about 10% of all patients with diabetes. The pathogenesis includes genetic and environmental factors [1]. The disease is preceded by a pre-diabetic period with progressive beta cell destruction and formation of islet related autoantibodies [2]. Histological analysis of post mortem specimens from pancreas donors did not reveal insulitis in individuals with islet autoantibodies [3]. In contrast, in newly diagnosed T1D patients beta cells may be present and various degree of insulitis with infiltration of macrophages and CD4^+^ and CD8^+^ T-cells is seen [4], [5]. At the final stage islets are devoid of beta cells and inflammatory infiltrates.

IAPP [6], [7], is a beta cell hormone, secreted together with insulin upon glucose stimulation [8]. Over the years, IAPP has been ascribed a wide range of biological functions, most of which are involved in glucose homeostasis. Identification of IAPP-receptors on beta cells [9], point to an auto- or paracrine function for IAPP. Increased insulin secretion in IAPP deficient mice in response to an oral glucose load supports an intra-islet function [10]. Also infusion of an IAPP-specific receptor antagonist during a hyperglycemic clamp augmented insulin secretion in parallel with a proportional increase in glucose disposal rate [11]. In a patient with a malignant pancreatic tumour circulating IAPP was determined to be 400 times higher than normal basal IAPP levels. Metabolic characterization of the patient showed that insulin secretion was fully blocked while the peripheral insulin sensitivity remained unaffected [12].

IAPP-amyloid is present in the islets of Langerhans in almost all individuals with type 2 diabetes, but is also seen in other conditions associated to beta cell stress, such as islet transplantation [13]. The complete pathway for protein misfolding needs to be identified but high IAPP concentrations are believed to be one factor important for initiation of aggregation. Amyloid fibrils are formed via smaller intermediates often referred to as oligomers or protofibrils, and the general perception is that certain oligomeric species are cytotoxic, and therefore is the formation of amyloid fibrils is considered to be more harmful than the deposited amyloid itself [14], [15]. However, growing amyloid deposits will interfere with cell-cell signalling and nutritional transport.

It is unknown whether IAPP-aggregation has any function in the development of T1D. One can assume that during beta cell destruction that precedes T1D, remaining beta cells are exposed to an increased functional demand similar to that in type 2 diabetes. Therefore, the aim of this study was to determine if IAPP levels were linked to decreased C-peptide levels seen in T1D.

## Results and Discussion

### Plasma analyses

**Figure 1:**
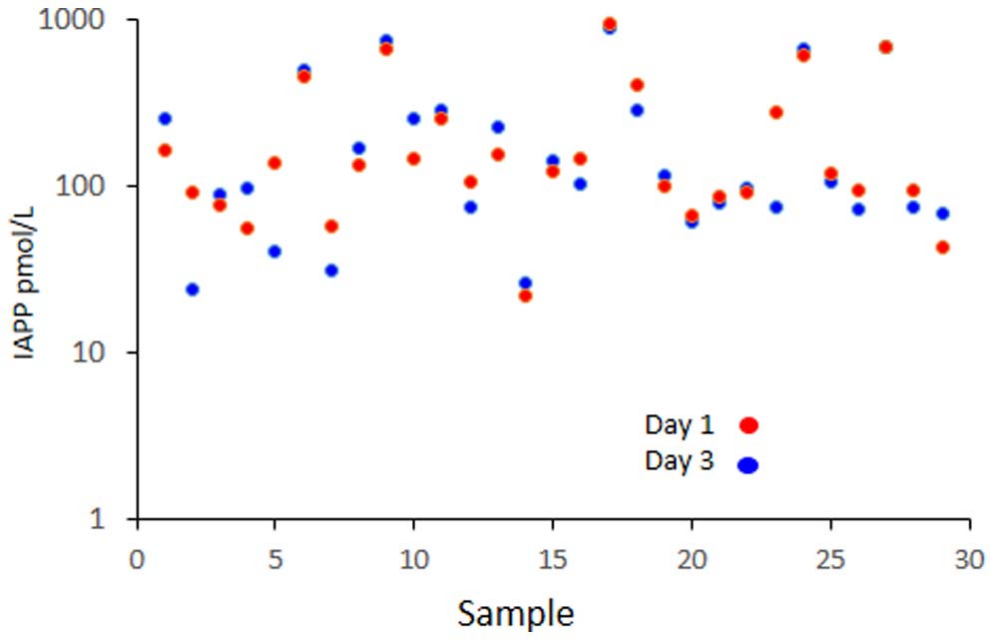
Elevated IAPP values in Day1 samples remain elevated in Day 3 samples. Comparison of IAPP concentrations in plasma taken at diagnosis (day 1) and three days after diagnosis (day 3). Red circles designate day 1 samples and blue circles day 3 samples.

This work was performed on plasma and serum samples from the Better Diabetes Diagnosis (BDD) study that aims to improve classification of diabetes in children and adolescents. This is a nationwide Swedish prospective cohort study that since 2005 recruits new-onset T1D children who are less than 18 years old at time of diagnosis. The diagnosis of T1D is established according to the American Diabetes Association. More than 2700 children were enrolled in the BDD-study between 2005 and August 2009, and out of these we selected the first 224 patients. Plasma samples from 30 healthy children, age 8–12 years were included as control group. All samples were taken at non-fasting condition. IAPP was analysed in samples taken day 1 (at diagnosis) and levels that exceeded 100 pmol/L were regarded to be high. This cut-off level exceeded the concentration determined in the control group (17.7±26 pmol/L; range 1–90). IAPP concentrations exceeding 100 pmol/L were detected in 25 subjects (11%). The determined levels ranged between 127.3 and 888.7 pmol/L (median 268.6) with the following distribution 10<200, 9<400, 2<600, and 4<900 pmol/L. IAPP levels in the remaining 199 samples ranged between 0 and 98.9 (pmol/L (median 4.8) (table 1). The IAPP ELISA standard curve ranges from 1.56 to 100 pmol/L. Samples exceeding 100 pmol/L were diluted and reanalysed. Henceforth, the groups with IAPP concentrations above 100 pmol/L and below 100 pmol/L are referred to high IAPP and low IAPP groups, respectively. IAPP analysis was repeated in plasma taken day 3 from subjects (n = 29), and the determined concentrations were found to be consistent with day 1 (figure 1).

**Table 1:** Hormone levels determined in plasma in newly diagnosed type 1 diabetes.

	Low IAPP	High IAPP		Control Plasma
IAPP (pmol/L)	3.4	268.5	p<0.0001	4.5
C-peptide (nmol/L)	0.21	0.19	p = 0.4	
Proinsulin (pmol/L)	11.6	10.6	p = 0.5	
Glucagon (pg/ml)	100	100	p = 0.4	

Values are given as median

To exclude methodological problems behind the elevated IAPP measurements we have carefully evaluated the procedure. According to the manufacturer, analyses of samples with hemolysis and lipidemia can give false result, but after examination of our samples we can exclude both hemolysis and lipidemia as a cause for the elevated IAPP values. Also, detection of comparable IAPP concentrations in samples taken day 1 and 3 argues against variations in individual samples. IAPP belongs to the calcitonin family and shares sequence similarities with calcitonin, calcitonin gene related peptide and adrenomedullin, but according to the manufacture the reported cross-reactivity with these peptides is < 1% in this ELISA. The IAPP analyses were performed with assays purchased at different time points and the results are not derived from analyses performed with plates from a single batch.

There are earlier reports on unexpected results in plasma from individuals diagnosed with T1D that depend on heterophilic antibodies [16]. Heterophilic antibodies are naturally occurring antibodies known to interfere with immunoassays and produce serious errors. This is especially true for “sandwich” ELISA systems where the capture and detection antibodies are from the same species. In this type of system heterophilic antibodies can cross-link the capture and the detection antibody. The ELISA used for IAPP measurements uses murine antibodies for capture and detection of IAPP. It is known that addition of murine immunoglobulin will block the undesirable binding of heterophilic antibodies and this is an accepted procedure [17]. According to the information given by the supplier murine IgG is included in the capture antibody solution. Heterophilic antibodies are low affinity antibodies and dilution of a sample containing heterophilic antibodies will reduce their binding in a non-linear fashion [18]. We have performed multiple dilutions of plasma from individuals with high IAPP levels and repeated the analyses but the results thereof revealed a linear relationship between dilution and IAPP concentration. Based on the above results we exclude cross-reactivity with IAPP related peptides or heterophilic antibodies as a cause for the determined high IAPP concentrations.

**Figure 2:**
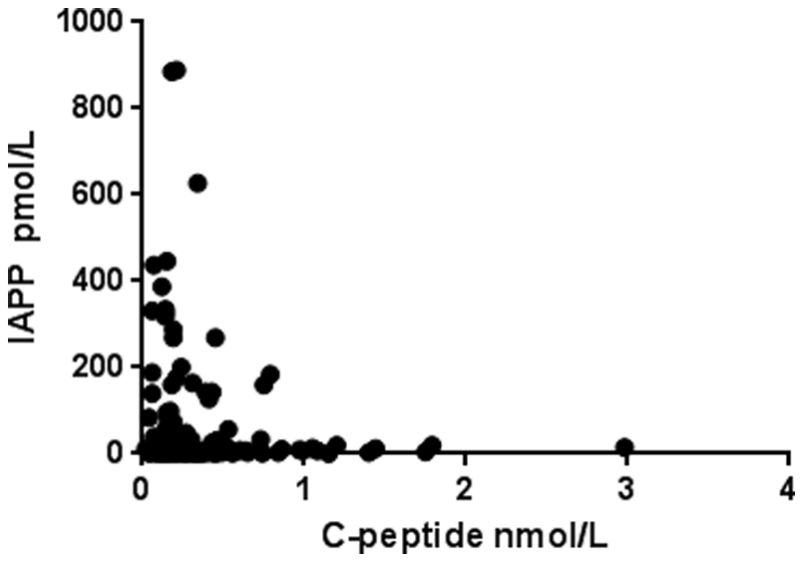
Comparison of IAPP and C-peptide concentrations in plasma from young with newly diagnosed T1D.

Serum C-peptide concentrations were determined to 0.19 nmol/L (0.04–0.79) in the high IAPP group and to 0.21 nmol/L (0.02–2.98) in the low IAPP group (p = 0.4). Comparison of IAPP and C-peptide concentrations did not reveal any association (r = −0.075) (figure 2). The proinsulin concentration was determined to 10.6 pmol/L (2.1–71.5) in plasma of the high IAPP group vs 11.6 pmol/L (2.8–101.7) in plasma of the low IAPP group, (p = 0.5). The plasma glucagon concentration was 100 pg/ml (50–190) in the high IAPP group and 100 pg/ml (50–400) in the low IAPP group (p = 0.4) (table 1).

### Analysis of clinical data

We analysed the collected clinical data for differences between the high and low IAPP concentration groups but neither of these analyses revealed any differences. Thus, the age at diagnosis was 11±4 years in the high IAPP group which did not differ from 10±4 years in the low IAPP group, (p = 0.6). In the high IAPP group 36% were girls and 16% of the subjects in this group were overweight while in the low IAPP group 45% were girls and 9% of the subjects were overweight. The blood glucose concentration was 25.2±6 mmol/L and 27 ±7 mmol/L (p = 0.2), and the HbA1c 10.3±2.5% and 10.1±2.6%, (p = 0.3), in the high and low IAPP concentration groups, respectively (table 2).

**Table 2:** Clinical data determined in newly diagnosed type 1 diabetes.

	Low IAPP	High IAPP	
Age at diagnosis (years)	10±4	10±6	p = 0.9
Blood Glucose (mmol/L	26±7.5	27±6	p = 0.2
HbA 1c (%)	10.0±2.5	10.6±2.7	p = 0.34
Sex (girls %)	48	28	
Overweight (%)	11.2	9.8	

Values are given as mean ± SD.

### Analysis of plasma for IAA autoantibodies

**Figure 3:**
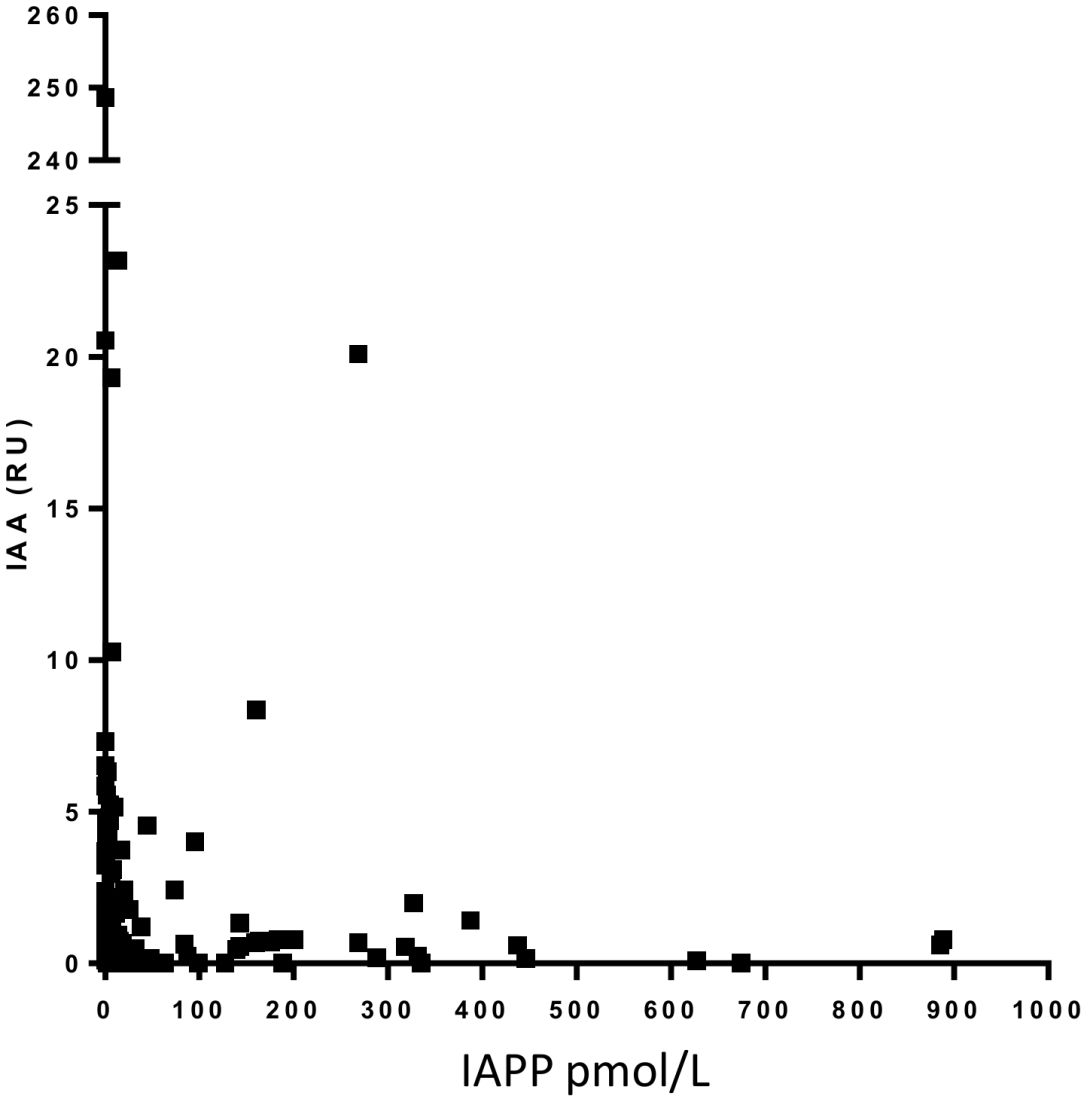
There is no correlation between presence of insulin autoantibodies and plasma IAPP concentration. Analysis of insulin autoantibodies in plasma from children and adolescent newly diagnosed with T1D.

A variety of autoantibodies have been described associated with T1D and the majority of these are directed against intracellular antigens [19], [20]. Presence of insulin autoantibodies (IAA) were analysed in plasma with a radiobinding assay [21]. IAA were identified in two samples from the high IAPP group and in five samples from the low IAPP group. There was no correlation between high IAPP concentrations and IAA levels (figure 3).

### Analysis of plasma for IAPP autoantibodies

**Figure 4:**
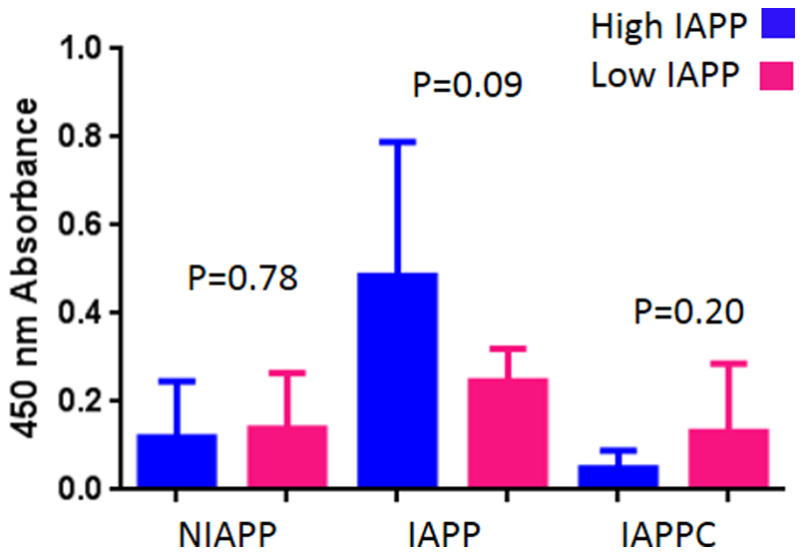
Analysis of autoantibody reactivity against NIAPP, IAPP and IAPPC in plasma from newly diagnosed T1D. High IAPP values are represented by blue bars and low IAPP values are represented by purple bars.

We speculated if autoantibodies against IAPP could give rise to the high IAPP values, and established an ELISA for analysis of proIAPP/IAPP autoantibodies. For this, peptides NIAPP, IAPP and IAPPC covering the complete length of proIAPP (see material and methods) were solubilised in dimethyl sulfoxide (1% final concentration) and diluted with 0.1 M carbonate buffer, pH 9.8. Final peptide concentrations were 0.1 μg/μl and 100 μl was used to coat each well of a 96-well plate. This should lead to the exposure of a large variety of proIAPP and IAPP related secondary structures. Circulation antibodies, determined in this way in plasma from 7 high (159, 188, 287.9, 331, 437.2, 625.1 and 888.7 pmol/L) and 6 low IAPP (2.1, 3, 6.9, 16.7, 20.3 and 38 pmol/L) individuals did not differ between the two groups (NIAPP p = 0.78, IAPP p = 0.09 and IAPPC p = 0.20 (respectively) (figure 4). With the use of ELISA, IAPP autoantibodies were detected in 18% of analysed plasma samples from patients with T1D [22]. However, if autoantibodies against human IAPP would contribute to the high IAPP concentrations cross-reactivity must occur between the murine capture antibody and human immunoglobulins. It should be pointed out that the high frequency of IAPP autoantibodies in T1D has been contradicted [23]. Also, when a radiobinding assay was used, that is considered to be more specific than ELISA, IAPP-autoantibodies could only be detected in 3 out of 65 T1D plasma samples [24]. We failed to detect any binding to NIAPP, IAPP and IAPPC with plasma from high or low IAPP groups. Therefore, the high IAPP concentrations found in the present study are unlikely to result from the presence of autoantibodies against IAPP.

Detection of high IAPP plasma concentration in 25 of the 224 tested samples was unexpected, since these patients had developed clinical T1D. Increased IAPP plasma concentration and amyloid formation has been shown to be associated with type 2 diabetes, but we found high IAPP levels in children and adolescents with classic T1D, which develops when less than 10% of the beta cell function remains [25]. The co-storage of IAPP and insulin in the secretory granules normally results in a simultaneous release of the two polypeptide hormones and they are expressed in parallel in the beta cells in response to glucose [26],[27]. Thus a decrease in insulin secretion would be expected to be accompanied by a decrease in IAPP release. We found no correlation between high IAPP levels and high BMI. Neither did we find any correlation to severity of diabetes (blood glucose, HbA1c) or other islet hormones such as proinsulin or glucagon, reflecting islet cell function.

It has been reported that beta cells depleted of extracellular calcium secrete minimal amounts of insulin both at 1.67 and 16.7 mmol/L glucose in contrast to IAPP where the glucose stimulated secretion was not altered by calcium depletion [28]. The secretion through the regulated pathway is blocked in the absence of calcium while the constitutive pathway would remain unaffected [28]. IAPP may therefore be secreted both through the regulated and the constitutive pathway and secretion of IAPP and insulin may not always be coupled. This may be of importance as insulin in a strong inhibitor of the aggregation of IAPP into toxic assemblies [29].

One could ask if IAPP produced outside the pancreas could be responsible for the high plasma concentrations seen in our T1D patients. In man, pancreas has been shown to be the main source for IAPP but there are also IAPP secreting cells in the pyloric antrum [30]. To what degree this rather limited number of cells could be the source and contribute to the plasma levels of IAPP is unknown, but most likely the cell number is not sufficient.

In summary, while high IAPP expression and amyloid formation so far has been seen in type 2 diabetes, we show that a quite substantial group of children and adolescents with classic T1D have high IAPP concentrations in plasma at the clinical onset of disease. The analysed samples in our study represent the onset of the clinical disease, even though the autoimmune disease process may have lasted for long time. High concentration of IAPP increases the risk for cell toxicity [31], [32] and amyloid formation [33], particularly in the absence of insulin. Therefore we speculate that the marked increased IAPP concentrations may constitute a risk factor for beta cell destruction also in T1D. It is possible that the group with high IAPP plasma concentration could represent a subgroup within T1D subjects. Further studies of the possible importance of IAPP for development of T1D are obviously highly needed.

## Materials and Methods

Plasma samples were taken at diagnosis, (day 1) and 3 days later, (day 3) and kept at −80°C, until use. Information on blood glucose, HbA1c, weight, age and sex were taken from the BDD records in collaboration with the childhood diabetes register SWEDIABKIDS. The BDD and this study have been approved by the Research Ethics Committee at the Karolinska Institute, Stockholm and all parents have given their oral and written informed consent. Plasma samples from 30 health children were retrieved from the plasma archive at Linköping University.

### Plasma measurements

IAPP levels were determined in plasma with a human IAPP ELISA kit (Linco, Research, Inc. St Charles, Mo., USA), plasma proinsulin levels with a human proinsulin ELISA kit (Mercodia, Uppsala, Sweden) and glucagon concentration with a human glucagon radio immuno assay (Linco). C-peptide was measured in serum samples with a time-resolved fluoroimmunoassay (AutoDELFIA™ C-peptide kit, Wallac, Turku, Finland) as the study progressed. The detection level was 0.03 nmol/L. Each assay was validated by inclusion of a C-peptide control module containing a high, a medium and a low-level control (Immulite, DPC, UK). A 1224 MultiCalc progra (Wallac) was used for automatic measurement and results calculation. All samples were analysed in duplicates at Linköping University. IAA measurements in plasma were performed with radiobinding assay at Lund University.

### Analysis of plasma of IAPP autoantibodies

Peptides corresponding to human N-terminal flanking peptide with IAPP ((NIAPP), residues 1–51), IAPP (residues 12–49 of proIAPP) and IAPP with the C-terminal flanking peptide ((IAPPC), residues 12–67 of proIAPP) were expressed and purified as described earlier [34]. Lyophilized NIAPP (1 mg), IAPP (1.2 mg) and IAPPC (1.1 mg) were solubilized in 100 μl dimethyl sulfoxide (Sigma, St Louis, MO, USA), diluted with 10 mlof 0.1 M carbonate buffer, pH 9.8, and an aliquot of 100 μl was added to each well of 96-well plates (Immunolone 2HB plates, Thermolab systems, Franklin, MA, USA). Plates were incubated over night at room temperature. Unbound peptides were washed out and remaining free binding sites were blocked with 3% albumin for 2 hours. Human plasma with IAPP concentrations above 120 pmol/L (n = 7) or with IAPP concentrations below 30 pmol/L (n = 6) were diluted 1∶100 and 1∶200 in 0.05 M tris-HCl, pH 7.4, with 0.15 NaCl (TBS). Each sample and dilution was analysed in triplicate and a volume of 100 μl was added to each well and the plates were incubated overnight, at 4°C. Six wells on each plate were incubated with secondary antibody only and used for background control. The presence of autoantibodies against IAPP or the processing intermediates was visualized with rabbit anti-human IgG antibodies labelled with horseradish peroxidase (Dako, Glostrup, Denmark), diluted 1∶1500 in TBS and 3,3,′5,5′ tetrametylbenzidine (Sigma). Optical densities were measured at 450 nm in a FLUOstar Omega, (Labtech, Offenburg, Germany) and data presented as median value of each sample.

### Statistical analysis

Values of hormone levels are given as median and range, clinical data are given as mean ± SD, and absorbance for autoantibodies are given as median. The data were analysed statistically with Mann-Whitney non-parametric test and Pearson correlation (GraphPad Software, San Diego, CA, USA) and a p-value < 0.05 was regarded as significant.
